# Long-term effectiveness and predictors of success of low-intensity shockwave therapy in phosphodiesterase type 5 inhibitors non-responders

**DOI:** 10.1080/2090598X.2019.1688072

**Published:** 2019-11-11

**Authors:** Zubairu S. Musa, Ahmed El-Assmy, Ahmad M. Shokry, Ahmed A. Shokeir, Tamer Zween, Mahmoud R. Al-Kenawy

**Affiliations:** a Department of Urology, Urology and Nephrology Center, Mansoura University, Mansoura, Egypt; b Department of Radiology, Urology and Nephrology Center, Mansoura University, Mansoura, Egypt

**Keywords:** Phosphodiesterase type 5 inhibitors, erectile dysfunction, low-intensity shockwave therapy

## Abstract

**Objective**: To evaluate the long-term (18 months) effectiveness, safety, and factors that may predict the success of low-intensity shockwave lithotripsy (Li-SWT) in patients with erectile dysfunction (ED) who fail to respond to oral phosphodiesterase type 5 inhibitors (PDE5i).

**Patients and Methods**: This prospective study included 52 patients with ED of vascular origin who failed to respond to oral PDE5i. The International Index of Erectile Function-Erectile Function domain (IIEF-EF) and Erection Hardness Score (EHS) questionnaires were used to evaluate EF. Patients under went two Li-SWT treatment sessions per week for 3 weeks, followed by a 3-week treatment-free period, and the cycle was repeated until each patient received 12 treatment sessions. Patients were followed-up after Li-SWT at 3, 6, 12, and 18 months.

**Results**: At the 18-month follow-up, 33 patients (63.5%) were able to achieve an erection sufficient for penetration with or without PDE5i (22 were maintained on oral PDE5i). The remaining 19 patients (36.5%) had a poor response to Li-SWT and oral PDE5i. The initial response showed some decline in 50% of the initial responders. Younger men (aged <45 years), short ED duration (<2 years), and moderate ED severity responded better to Li-SWT. There were no adverse side-effects.

**Conclusion**: In the present study, Li-SWT was a safe and effective treatment in 63.5% of men with ED who failed to respond to oral PDE5i. Factors such as age (<45 years), ED duration (<2 years), and ED severity can predict treatment outcome in such patients.

**Abbreviations:** CDU: colour Doppler ultrasonography; ED: erectile dysfunction; EDV: end-diastolic velocity; EF: erectile function; EHS: Erection Hardness Score; FU: follow-up; IIEF-EF: International Index of Erectile Function-EF domain; Li-SWT: low-intensity shockwave lithotripsy; PDE5i: phosphodiesterase type 5 inhibitors; PGE1: prostaglandin E1; PSV: peak systolic velocity; RI: resistive index; VOD: veno-occlusive dysfunction

## Introduction

Erectile dysfunction (ED) is the persistent/recurrent inability to attain or maintain an erection sufficient to permit satisfactory sexual intercourse. ED affects physical and psychological health, and may have a significant impact on the quality of life of men and their partners. There is increasing evidence that ED can be a potential warning sign of coronary artery and peripheral vascular diseases [1,2].

Non-surgical treatments for managing ED include: oral phosphodiesterase type 5 inhibitors (PDE5i), vacuum erection devices, and intracavernosal injection of vasodilating agents. These treatments are relatively safe with few adverse effects; however, they share the same major setback in that they do not alter the under lying pathophysiology of the ED and their effects are essentially time limited.

Low-intensity shockwave therapy (Li-SWT) induces cellular microtrauma, which in turn stimulates the release of angiogenic factors with the subsequent neovascularisation of the treated tissue. These findings led to the assumption that if Li-SWT was applied to the penis it could improve blood supply to the corpora cavernosa and improve erection. Many studies have been conducted assessing the safety, feasibility and effectiveness of penile Li-SWT for treating ED or converting men with ED who failed to respond to oral PDE5i [3–5].

But to the best of our knowledge, no study has been carried out to assess the factors that could affect or predict ED Li-SWT treatment success in men who are PDE5i poor responders. Also, there are very few articles that have discussed long-term outcomes of penile Li-SWT. This prompted us to assess the long-term outcome (at 18 months) of Li-SWT in men with vasculogenic ED who failed to respond to PDE5i, and evaluated factors that might influence or predict Li-SWT success amongst such patients.

## Patients and methods

### Study population

This prospective study comprised 55 patients with vasculogenic ED who failed to respond to PDE5i and presented to our male andrology outpatients clinic. Patients were considered PDE5i non-responders after completing all optimisation measures (e.g., correct dose optimisation of PDE5i, correction of risk factors, improvement in sexual stimuli, correction of testosterone levels) and still manifesting with an inability to achieve vaginal penetration (Erection Hardness Score [EHS] ≤2) [4].

The study protocol was reviewed and approved by our local Ethics Committee. All participants signed a written informed consent before entering the study. The study was conducted between January 2015 and October 2017. Inclusion criteria included: aged 20–80 years, patients with ED of >3-months duration, stable marital and sexual relationship with the same partner of >3 months, and negative response to PDE5i with failure of vaginal penetration. While any cause of ED other than vasculogenic, e.g., hormonal, neurological, psychiatric problems, and anatomical penile abnormalities, were excluded.

### Pre-treatment evaluation

A detailed history was taken and included: age; marital status; and social habits, such as cigarette smoking and alcohol consumption. Medical history and related co-morbidities such as hypertension, diabetes mellitus (DM), hyperlipidaemia, cardiovascular diseases, chronic renal insufficiency, chronic liver disease, thyroid disorders, hypogonadism and immunosuppressive diseases, were documented for each patient. Medication history was taken, with focus on the use of anti-hypertensives, anti-androgens, anti-psychotics, anti-depressants, and opiates intake. Surgical history was taken, where attention was paid to any history of pelvic trauma, pelvic/perineal surgeries, urethral interventions, and exposure to pelvic radiotherapy. Sexual history was obtained using the International Index of Erectile Function-Erectile Function domain (IIEF-EF) [6] and EHS [7] questionnaires. All patients under went a 1-month washout period of PDE5i before starting Li-SWT.

General and systemic examinations were carried out including: weight, height, and body mass index (BMI). Baseline laboratory investigations were performed as follows: urine analysis, urine culture and sensitivity (when indicated), complete blood count, serum creatinine, liver function tests, and random blood sugar.

Penile colour Doppler ultrasonography (CDU) was used to assess penile haemodynamic changes. Penile CDU was carried out in a private quiet room; erection was induced by intracavernosal injection of 20 µg prostaglandin E1 (PGE1). To achieve complete cavernosal smooth muscle relaxation, patient self-stimulation was encouraged. Penile CDU (Accuson-128 XP; Accuson Inc., Mountain View, CA, USA), with linear transducer (5–10MHz), was performed within a minute of the intracavernosal injection of PGE1. Scanning was started at the base of the penis, the cavernosal arteries were identified with serial measurement of peak systolic velocity (PSV), end-diastolic velocity (EDV) and resistive index (RI) every 5 min for 30 min, until maximum PSV and minimal EDV were reached. A PSV of <30 cm/s was considered as indicative of arterial insufficiency, a RI <0.85, and EDV >5 cm/s were considered as suggestive of veno-occlusive dysfunction (VOD) [8].

### Li-SWT treatment

For Li-SWT we used a Dornier Aries shockwave device (Dornier Med Tech GmbH, Weßling, Germany) measuring 20 × 25 × 40cm (length, width and height).

The patients had two sessions of Li-SWT per week. During each session 1500 shocks (energy density 0.009 mJ/mm^2^) were applied at five different penile areas; three along the penile shaft (upper, middle and lower aspects) and two at the left and right crura. Shockwaves were delivered through an applicator covering the corpora cavernosa of the penis along the penile shaft and the crura [9]. The patients had two treatment sessions per week for 3 weeks, followed by a 3-week treatment-free period. The cycles were repeated until patients’ received 12 total treatment sessions. No anaesthesia was given.

### Follow-up

Patients were followed up after Li-SWT at 3, 6, 12 and 18 months. During each follow-up the patients completed the IIEF-EF domain and EHS questionnaires.

### Outcome measures

At 3 months patients were evaluated and classified into:

1. Li-SWT responders, when patients reported successful sexual intercourse with an erection sufficient for penetration (EHS ≥3). Those patients were followed-up to 6, 12, and 18 months.
2. Li-SWT non-responders, when patients reported weak erection not sufficient for vaginal penetration (EHS <3). The non-responders were offered oral 50 mg daily sildenafil citrate tablets for 3 months and then re-evaluated for further subdivision in to:- (a) PDE5i converters and (b) PDE5i non-converters

We evaluated patients at 18 months for the long-term outcomes of Li-SWT; treatment success at 18 months was defined as an EHS ≥3 with or without the use of oral PDE5i. Also at this time point, we evaluated factors that may have influenced treatment success.

### Statistical analysis

The data were collected and statistical analyses performed using the IBM Statistical Package for the Social Sciences (SPSS®), version 20 (SPSS Inc., IBM Corp., Armonk, NY, USA). Categorical variables were compared using chi-squared and continuous variables were compared using unpaired *t*-tests. A *P *≤ 0.05 was considered to indicate statistical significance.

## Results

A total of 55 patients were enrolled. Three patients were lost to follow-up and were excluded from the study; hence, only 52 patients completed the 18-month follow-up and were included in the final analysis. Baseline laboratory investigations were normal except in four patients who had pus cells in the urine, which yielded no growth of microbes and they were treated with oral ciprofloxacin (500 mg 12-hourly for 10 days). The patients’ demographic data are shown in Table 1.

**Table 1.** Patients’demographics.

Variable	Value
Medical co-morbidity, *n*	
Hypertension	16
DM	14
Hypertension and DM	15
Relevant surgical history, *n*	
Transurethral surgery (TURP, BNI, TUIP)	6
Multiple transurethral surgeries	1
Smoking, *n*	21
Ages, years, mean (SD; range)	51 (11.56; 23–74)
Duration of ED, months, mean (SD; range)	34.5 (1.18; 9–60)
Baseline IIEF score, points, mean (SD; range)	11 (2.8; 6–16)
Baseline EHS, points, mean (range)	2 (1–2)
BMI, kg/m^2^, mean (SD; range)	27.46 (2.8; 21.8–36.3)

BNI: bladder neck incision; TUIP: transurethral incision of the prostate.

### At the 3-month follow-up

In all, 22 patients (42.3%) reported successful satisfactory sexual intercourse (EHS ≥3) without PDE5i (Li-SWT responders). The remaining 30 patients (57.7%) had no successful satisfactory vaginal penetration. Amongst those 30 patients, 21 patients had increased IIEF-EF scores of >5 points. The 30 Li-SWT non-responder patients were placed on oral PDE5i and were followed-up at 6-months.

### At the 6-month follow-up

The 22 patients who initially had an EHS ≥3 without PDE5i (Li-SWT responders) sustained their response. Of the 30 patients who were given oral PDE5i, 11 patients (21.1%) became PDE5i converters (had EHS ≥3) and the remaining 19 patients still have unsatisfactory sexual intercourse (PDE5i non-converters). All the 11 patients who became PDE5i convertors had an initial increased IIEF-EF score of >5 points after Li-SWT.

### At the 12-month follow-up

Three patients (5.8%) of the 11 PDE5i converters showed a marked improvement in IIEF-EF and had good satisfactory sexual intercourse without the need of PDE5i, and the remaining eight PDE5i converters sustained their response. The 19 patients who were PDE5i non-converters still had unsatisfactory sexual intercourse. Again, the 22 patients who had an EHS ≥3 (at the 3- and 6-month follow-ups) without PDE5i (Li-SWT responders) sustained their response

### At the 18-month follow-up

The 11 patients (50%) of the 22 Li-SWT responders who had an initial good response to Li-SWT and could practice intercourse without the need of oral PDE5i had some levels of decline in their response to Li-SWT but were able to use PDE5i successfully. At the last follow-up, 33 patients (63.5%) had a good/satisfactory sexual life, 22 of them had to be maintained on oral PDE5i. The remaining 19 patients (36.5%) had a poor response to Li-SWT and oral PDE5i.

We investigated the factors that predicted success of Li-SWT (EHS ≥3, with or without PDE5i) in our patients at the 18-month follow-up, and we found that younger patients with a short ED duration and a moderate degree of ED responded better to Li-SWT (Table 2). Additional study of these factors identified threshold values of 45 years and 2 years for the age and ED duration, respectively, according to the receiver operating characteristic curve.

**Table 2.** Factors that may influence Li-SWT outcome.

Variable	Responders	Non-responders	*P*
Number of patients	33	19	
Age, years, mean (range)	46.5 (28–65)	62.5 (41–74)	0.031
ED duration, month, mean (range)	22.5 (9–36)	36.0 (12–60)	<0.001
ED severity, *n* (%)			0.02
Severe	8 (24.3)	14 (73.7)	
Moderate	25 (75.7)	5 (26.3)	
Hypertension, *n* (%)	10 (30.3)	6 (31.5)	0.66
DM, *n* (%)	9 (27.2)	5 (26.3)	0.71
Hypertension+ DM, *n* (%)	9 (27.3)	6 (31.5)	0.27
DM duration, months, mean (range)	33 (12–60)	32 (10–60)	0.28
Smoking, *n* (%)	13 (39.4)	8 (41.1)	0.34
Transurethral surgery, *n* (%)	4 (12.1)	3 (15.7)	0.54
BMI, kg/m^2^, mean (range)	27.6 (20.12–31.5)	29.94 (26.32–36.36)	0.61
Penile CDU findings, *n* (%)			0.83
VOD	28 (85)	18 (94.7)	
Arteriogenic	5 (15)	1 (5.3)	

## Discussion

Penile Li-SWT has recently emerged as a novel and promising modality in the treatment of ED. Unlike other current treatment options for ED, all of which are palliative in nature, Li-SWT is unique in that it aims to restore the erectile mechanism in order to enable natural or spontaneous erections [4]. When Li-SWT is applied to an organ, the shockwaves interact with the targeted tissues and induce a cascade of biological reactions, which stimulate release of growth factors, which in turn triggers neovascularisation of the tissues with subsequent improvement in the blood supply [10].

In a series of clinical trials, including randomised double-blind sham-controlled studies, Li-SWT has been shown to have a substantial effect on penile haemodynamics and EF in patients with vasculogenic ED, without any adverse effects [3–5,11-13].

Vardi et al. [3] in 2012 reported the first randomised, double-blind, sham-controlled study that showed that Li-SWT has a positive clinical and physiological effect on the EF of men who were PDE5i responders. They found a significantly greater increase in the IIEF-EF domain score and improved penile haemodynamics after 1 month in the Li-SWT group than in the sham-treated group. In a meta-analysis published in 2017, which reviewed 14 studies including 833 patients, it was reported that Li-SWT could significantly improve IIEF and EHS, and therapeutic efficacy could last at least 3 months. The patients with mild and moderate ED had better therapeutic efficacy after treatment than patients with more severe ED or comorbidities [14]. The most recent meta-analysis in 2019, which evaluated 10 randomised controlled trials including 873 patients, showed that Li-SWT significantly improved EF in patients with vasculogenic ED [15].

Li-SWT is not only effective in patients who are responsive to PDE5i but can also convert PDE5i non-responders to responders. The first double-blind, sham-controlled study that evaluated Li-SWT in the treatment of patients unable to achieve sexual intercourse using PDE5i was reported by Kitreuy et al.  [12] in 2016. In their study, 58 patients were randomised including 37 treated with Li-SWT and 18 treated with a sham probe. In the sham group, 16 patients underwent Li-SWT treatment 1 month after sham treatment. In the Li-SWT treatment group and the sham group 54.1% and 0% of patients, achieved erection sufficient for vaginal penetration, respectively. Of patients subsequently treated with Li-SWT after sham treatment, 56.3% achieved erection sufficient for penetration. However, that study had several limitations; the number of patients was relatively small and the follow-up was short. The Li-SWT effect was evaluated only during obligatory PDE5i treatment and therefore the proportion of patients who could achieve satisfactory erection without PDE5i was not clear.

A similar rate of success to that of the previous study was reported in another prospective series, which included only 20 men with ED who failed to respond to oral PDE5i. The treatment consisted of four sessions over a 4-week period, during each session the patient received 5000 shockwaves; 1800 were applied on the penis and 3200 were applied on the perineum. During the active treatment and follow-up phases, all patients remained on their regular high on-demand or once-a-day dose PDE5i schedules. In all, 60% of the patients responded to the treatment [13].

Our present findings are consistent with the two above mentioned studies, in that Li-SWT was effective in the treatment of ED in men who had failed to respond to PDE5i as evidenced by successful vaginal penetration in 63.5% of our patients with or without use of PDE5i. The advantage of our present study is that we stopped oral PDE5i before Li-SWT, so that we could ascertain the percentage of men who would regain their potency without the use of oral PDE5i. At 18 months; only 11 men (21.1%) had an EHS >3 without oral PDE5i. Additionally, none of the above studies including our present study has reported any adverse effect of Li-SWT.

The previous studies mainly focused on the effectiveness and safety of Li-SWT in the treatment of vasculogenic ED in patients who failed to respond to oral PDE5i. We found that younger patients (<45 years), with short ED duration (<2 years), and moderate ED responded better to Li-SWT than older patients with severe ED and long ED duration. These factors could be used to help the urologist in selecting patients who could get the maximum benefit from Li-SWT and avoid disappointment in those where Li-SWT is likely to be ineffective. These factors may help us to choose other lines of treatments, such as intracavernosal injection or penile prosthesis, for patients who do not wish to try a doubtful line of treatment.

An important question for the patients is for how long will the effects of Li-SWT last? The 12-month results of the effectiveness and safety of Li-SWT in patients with ED who were non-responders to PDE5i treatment was reported by Bechara et al. [16]. In their study, the Li-SWT treatment was effective and safe in 60% of their 50 patients and the efficacy response was maintained for 12 months in 91.7% of these patients. On the other hand, Kitrey et al. [17] studied the 2-year efficacy of penile Li-SWT after an initially successful outcome. They evaluated 156 patients who underwent the same treatment protocol but participated in different clinical studies. At 1 month, treatment was successful in 99 patients (63.5%). During follow-up a gradual decrease in efficacy was observed. The beneficial effect was maintained after 2 years in only 53 of the 99 patients (53.5%) in whom success was initially achieved. Patients with severe ED were prone to earlier failure than those with non-severe ED. During the 2-year follow-up, the effect of Li-SWT was lost in all patients with diabetes who had severe ED at baseline. Conversely, patients with milder forms of ED without diabetes had a 76% chance that the beneficial effect of Li-SWT would be preserved after 2 years.

Our present results are consistent with those of Kitrey et al. [17]. After 18 months, 11 of the 22 patients (50%) who had an initial good response to Li-SWT could still achieve intercourse without the need of oral PDE5i, and those who had some level of decline in their response to Li-SWT were able to use PDE5i successfully.

Finally, we acknowledge some limitations of our present study. Firstly, the sample size was small. Secondly, we did not randomise our present patients into treatment and sham groups. Finally, we relied on the patients’ assessment of their erection and we did not perform penile CDU at follow-up to assess haemodynamic changes.

## Conclusion

In the present study, Li-SWT was a safe and effective treatment in 63.5% of men with ED who failed to respond to oral PDE5i. After 18 months, the beneficial response showed some decline in 50% of responders but they were still able to use PDE5i successfully. Younger men (<45 years) with a short ED duration (<2 years) and moderate ED benefited more from Li-SWT than the older men with more severe and longer duration ED. However, there is a need for large scale multicentre controlled studies with long follow-ups to validate our present findings.
